# Generation of Controlled Micrometric Fibers inside Printed Scaffolds Using Standard FDM 3D Printers

**DOI:** 10.3390/polym15010096

**Published:** 2022-12-26

**Authors:** Elisa del Barrio Cortés, Clara Matutano Molina, Luis Rodríguez-Lorenzo, Nieves Cubo-Mateo

**Affiliations:** 1 Research Support Technical Unit, Aragon Health Research Institute Foundation, 50009 Zaragoza, Spain; 2 iBIO, Higher School of Science and Technology (ESICT), International University of Valencia, 46002 Valencia, Spain; 3 Nebrija Research Group ARIES, Higher Polytechnic School, Antonio de Nebrija University, 28040 Madrid, Spain; 4 Department of Polymeric Nanomaterials and Biomaterials, Institute of Polymer Science and Technology (ICTP), Spanish National Research Council (CSIC), 28006 Madrid, Spain

**Keywords:** 3D printing, microfibers, scaffolds, tissue engineering, polycaprolactone, printing parameters, algorithms

## Abstract

New additive manufacturing techniques, such as melting electro-writing (MEW) or near-field electrospinning (NFES), are now used to include microfibers inside 3D printed scaffolds as FDM printers present a limited resolution in the XY axis, not making it easy to go under 100 µm without dealing with nozzle troubles. This work studies the possibility of creating reproducible microscopic internal fibers inside scaffolds printed by standard 3D printing. For this purpose, novel algorithms generating deposition routines (G-code) based on primitive geometrical figures were created by python scripts, modifying basic deposition conditions such as temperature, speed, or material flow. To evaluate the influence of these printing conditions on the creation of internal patterns at the microscopic level, an optical analysis of the printed scaffolds was carried out using a digital microscope and subsequent image analysis with ImageJ software. To conclude, the formation of heterogeneously shaped microfilaments (48 ± 12 µm, mean ± S.D.) was achieved in a standard FDM 3D Printer with the strategies developed in this work, and it was found that the optimum conditions for obtaining such microfibers were high speeds and a reduced extrusion multiplier.

## 1. Introduction

One of the most challenging issues in the field of medicine has been to maintain, restore, or improve the function of damaged or lost organs and tissues in the human body. The major drawback of conventional treatments lies mainly in the difficulty of finding donors and the rejection of the transplanted organ/tissue by the recipient body. This is how the tissue engineering field was born in biomedicine, in order to develop functional tissues capable of regenerating and/or improving damaged tissue.

Tissue engineering requires the development of complex systems made of: (i) biomaterials for scaffold construction that should mimic the properties of the natural extracellular matrix [1], (ii) bioactive molecules such as growth factors that allow cells to multiply [2], and (iii) mesenchymal stem cells capable of differentiation [3].

This work deals with the engineering of biomaterials to produce cell-friendly scaffolds for tissue engineering. The main function of scaffolds is to provide a support system for cells, mimicking the cellular matrix in such a way that favors cell adhesion, proliferation, as well as cell growth [4]. Also, scaffolds should provide enough stability for the new tissue to form and the extracellular matrix to be deposited [5]. Both the mechanical properties and microscopic structure of the scaffolds will vary depending on the needs of the application. For example, nanofibrous scaffolds should be used for drug delivery on skin wounds [6] or water/air nanofiltration applications [7,8]. Therefore, controlling the macro and microscopic structure during fabrication is a suitable way to fulfill the requirements of the application and in the case of biomedical applications, it is a necessary step to enable and facilitate the tissue regeneration process.

The design of the scaffold structure at the macroscopic and microscopic level will fundamentally depend on its mechanical, physical, and molecular properties and will have a strong influence on the development of cell adhesion, proliferation, and growth [9].

For the creation of tissue engineering scaffolds, additive manufacturing techniques are employed to control the internal architecture and mechanical properties, resulting in highly reproducible constructions with known pore size and distribution [10]. However, the creation of scaffolds using 3D printing techniques is limited by the fact that the pore size produced is usually large for certain cellular systems and, therefore, correct cell adhesion and proliferation might not be guaranteed [11]. The recommended pore size for the scaffold to successfully fulfill its function is between 100–300 µm [12]. However, with 3D printing techniques the pore size obtained is usually between 300–700 µm [12], which is not adequate for cell adhesion and subsequent cell proliferation to occur.

**Figure 1:**
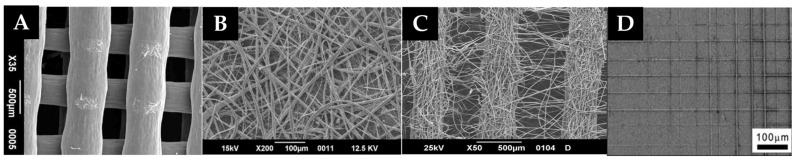
Scanning electron microscope images of: (**A**) Scaffold printed by FDM, (**B**) Scaffold printed by electrospinning, (**C**) Scaffold printed by hybrid technique (FDM + Electrospinning), (**D**) Scaffold printed by MEW. This figure is based on work by Sabino, M.A. et al. 2017 [3], licensed under CC BY 4.0.

To obtain the appropriate pore size, interconnectivity, and distribution, currently, 3D printing techniques are combined with conventional techniques such as electrospinning or melting electro-writing (MEW). This way, microscopic fibers will be formed between the pores that will favor cell migration and proliferation [13]. The main difference between the two techniques is that MEW can control the deposition of the microfibers, which makes it preferable for use in additive manufacturing. Another advantage of using MEW over electrospinning is that it is not necessary to remove toxic solvents usually used in electrospinning once printing is finished [14]. Through these techniques, microfibers between 5–30 µm can be created. The following subfigures (Figure 1) show the results of printing filaments using FDM, electrospinning, and MEW, for comparison.

Furthermore, by applying a combination of these techniques, it is possible to form macroscopic pores (by FDM) with microfibers between the pores (by MEW or electrospinning), thus simulating the extracellular matrix (ECM). The formed scaffold surface, including the microfibers, favors cell migration and proliferation [13].

Recently, a multi-technological approach allowed for the simultaneous combination of calcium phosphate cement (CPC) printing with MEW of polycaprolactone (PCL) microfibers in an alternating, tuneable design in one automated fabrication process [15]. The hybrid CPC+PCL scaffolds featured a strong interface and the microfiber integration led to an improvement in integrity. In addition, the incorporation of PCL fibers led to pore coverage by a human mesenchymal stem cell line and an elevated proliferation level of murine pre-osteoblasts, confirming what was previously noted concerning cell proliferation in microstructured environments, given the proper biomaterial [11,12].

Looking at other 3D printing technologies, the authors found a study that explores 3D printing stereolithography (SLA) to create nanosheets, spacers, or thin layers for water purification and surface imprinting [8]. However, this is excluded from the work presented here as it cannot be combined with the deposition of other biomaterials, hydrogels, and embedded cells.

Only one paper can be found where authors managed to print microfibers (<100 µm) by employing a commercial 3D extrusion printer [16]. However, they are vertical strands not combined with scaffold structures.

In summary, to introduce nano/microfibers into the body of a macroporous scaffold with the purpose of providing the topological cues suitable for cell proliferation and spreading, two main approaches have been used: the combination of techniques such as spinning methods or MEW, as described above [17,18,19], or the modification of the extruder [20,21]. Therefore, it can be concluded from the literature that sophisticated printing equipment and new extrusion methods are required for the fabrication of strands with a thickness smaller than 100 µm.

In a previous study carried out by one of the authors of this work evaluating the influence of printing parameters on the physical properties of scaffolds, it was pointed out that it was possible to obtain microfibers in a random manner via standard 3D printing [22]. Based on the information presented in that study, the hypothesis tested in this work was if it possible to achieve the creation of reproducible and controlled microscopic internal patterns in scaffolds by developing non-standard slicing algorithms for standard 3D printing, without the use of sophisticated printing equipment. This represents a novelty in the field as no similar work has been published before.

Therefore, the aim of this work is to provide and standardize a methodology to generate scaffolds with basic FDM machines that allows us to design its structure at the microscopic level using new deposition algorithms and strategically modifying some printing parameters in a controlled way. This will demonstrate that cheaper and more accessible additive manufacturing technologies can be used to fabricate scaffolds with microstructure infills.

## 2. Materials and Methods

**Figure 2:**
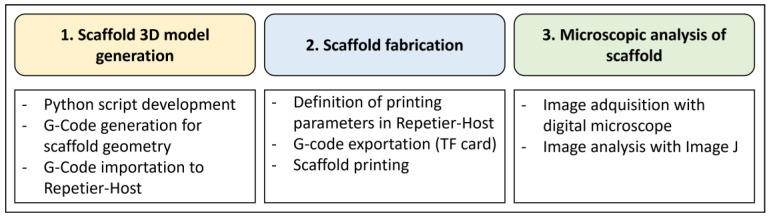
Stages of the methodology.

The methodology employed to evaluate microscopic internal patterns formed in standard 3D printed scaffolds was based on three stages, which are shown in Figure 2 and described in detail below.

### 2.1. Scaffold 3D Model Generation

In order to generate the 3D model of the scaffolds for subsequent printing, the creation of scripts in Python programming code was carried out, which allows for the automatic generation of the G-code required to perform the 3D printing. To this aim, Spyder 4.1.1.1 was selected as the ideal python programming platform to create the required scripts. The steps followed in this stage are detailed below.

#### 2.1.1. Creation of Scripts in Python Code for the Generation of the G-Code

**Figure 3:**
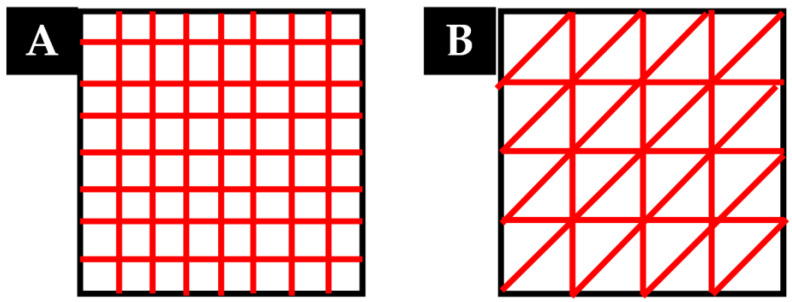
(**A**) Rectilinear geometric model, (**B**) Triangular geometric model. Red lines represent the extruder trajectories.

Initially, two scripts were developed in Python programming code with the objective of generating different geometries. These geometries are based on primitive geometric figures and correspond to the models shown in Figure 3. However, only the first was used for the entirety of this study. The second pattern was used only for the last subsequent study, in order to investigate the generation of microfibers via extruder passes without raising the layer in the Z-axis (Section 3.4).

Each script follows the following structure:

1. First, the independent variables are defined, including the scaffold dimensions (length, line spacing, layer height), the number of scaffold layers on the Z-axis, the printer nozzle diameter, the filament diameter, the printing speed, and the extrusion multiplier.

**Figure 4:**
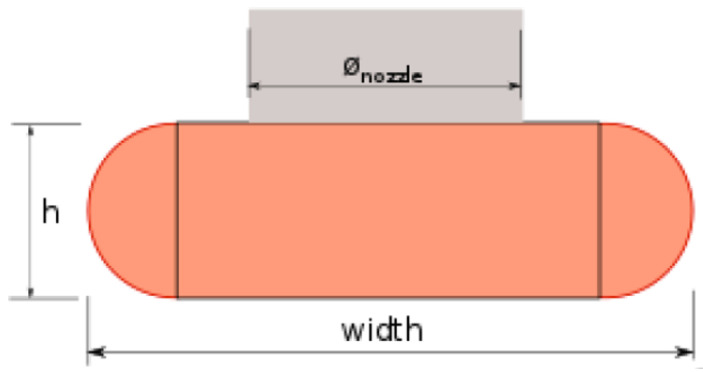
Extruded filament cross section showing the relationship between the desired extrusion width and volume to extrude. Reprinted from Slic3r Manual.

Subsequently, the dependent variables and corresponding equations are defined. It should be noted that to calculate the amount of extruded filament in each line of the scaffold (E), the equation used by the Slic3r software, which generates G-code from 3D CAD files, was employed. This equation considers that the extruded filament cross section is a rectangle with semi-circular edges, as shown in Figure 4 where:

- A is the cross section of the extruded filament (mm^2^)
- Ø, from now d, is the diameter of the printer nozzle (mm)
- h is the layer height (mm)
- w is the extrusion width (mm)

On the one hand, the volume of filament injected into the printer nozzle (Vin) can be obtained as follows:

(1) $$\mathrm{Vin}=\pi \;\times {\left(\frac{d}{2}\right)}^{2}\times \;E\;$$

where again:

- d is the diameter of the printer nozzle.
- E is the amount of extruded filament in mm.

On the other hand, the volume of filament required (Vout) to be able to print a line of width w can be calculated by multiplying the cross section of the extruded filament (A, given by the area of a rectangle plus a circle) by the length of the printed line (L):

(2) $$\mathrm{Vout}=A\;\times \;L=\left(\left(w-h\right)\times h+{\left(\frac{h}{2}\right)}^{2}\right)\times L$$

Therefore, taking into account that Vin = Vout, the amount of extruded filament (E) can be calculated with the following equation:

(3) $$E=A\;\times \;L\;\times 4\;/\left(\pi \;\times d^{2}\right)$$

Finally, it should be considered that the extruder multiplies the amount of extruded filament (E) by the extrusion multiplier (f) to obtain a suitable line width (w):

(4) $$E=\left(A\;\times \;L\;\times 4\;/\left(\pi \;\times d^{2}\right)\right))\times f$$

Once all the variables are defined, the code that allows for the generation of the coordinates in the XY plane and the reproduction of the scaffold geometry in the Z-axis is included.

2. Finally, the code that generates the G-code is defined, including the printing speed (instruction starts with F, followed by a number in mm/min), geometric coordinates (marked as X, Y and Z, followed by a number in mm), and the amount of extruded filament (marked as E and followed by a number in mm),\. It is worth mentioning that the value of E is cumulative.

As an example, the script for the rectilinear geometric model is attached in Appendix A of this document. A graph where the geometry created in the XY plane can be seen has been included.

#### 2.1.2. Generation of the 3D Model in Repetier Host

Once the G-codes with the trajectories were generated, they were imported into a 3D printer host that allow us to export it and proceed with the 3D printing. In this case, Repetier Host Mac V1.0.2 3D was the printing software selected to visualize and modify the G-code. Before proceeding to send instructions to the printer, the G-code must be modified manually to specify additional parameters not included in the trajectory algorithm such as extruder temperature, hot bed temperature, or fan speed, among others.

For more details on the G-code and the commands used, reference is made to Appendix B, where an example of the G-code created for printing a 1-layer scaffold using the rectilinear geometric model is shown.

### 2.2. Fabrication of Scaffolds

The fabrication of the scaffolds was carried out by standard 3D printing. In the following subsections, the steps carried out for their fabrication are described.

#### 2.2.1. Setting up the 3D Printer

A cartesian 3D printer Artillery Genius 2020 with the following technical specifications (Table 1) was used to print the scaffolds [23].

To obtain good printing quality of the scaffold, it is necessary to adjust the printing parameters. The most relevant printing parameters considered in the experiments carried out in this work are listed below:

3. **Layer height:** It is directly related to the printing Z-resolution. The higher the layer height, the shorter the printing time, but the worse the finish of the printed part. Generally, it is recommended not to use a layer height of more than 80% of the nozzle diameter of the printer [24]. That is, if the printer has a nozzle diameter of 0.40 mm, the layer height should not exceed 0.32 mm in any case.
4. **Extrusion width:** Thicker lines allow for better bonding between layers. However, it will worsen the accuracy of the shape. It is very important to choose the correct extrusion width, especially when printing the first layer to ensure good adhesion. Generally, values between 1.05 and 1.7 times the diameter of the printing nozzle are used [25].
5. **Print speed:** To obtain a good print, the optimum print speed values generally used are between 15 and 20 mm/s. The print quality will decrease at higher print speeds. Speeds above 100 mm/s may cause instability in the printer and, therefore, it is not recommended to print at higher speed values. It is also recommended not to print the first layer at speeds higher than 25 mm/s to avoid adhesion problems to the base [26].
6. **Extrusion temperature:** This will depend on the material used and will be defined according to the melting temperature of the material. If the extrusion temperature is determined to be above the melting temperature, too much filament will be extruded without control. Conversely, if the extrusion temperature is lower than the melting temperature of the material, no filament will be extruded. The optimum extrusion temperature value can be modified by other parameters such as printing speed [26]. The manufacturer of the materials used in this work recommends printing by setting an extrusion temperature between 130 and 170 °C for PCL [27].
7. **Hot bed temperature:** This is defined according to the material used and is essential to ensure correct adhesion to the printer base of the first printed layer. The value recommended by the material manufacturer is between 30 and 45 °C when working with PCL [27].
8. **Extrusion multiplier:** Its default value is 1 (100%). It can be modified to adjust the amount of extruded filament and the appropriate line width.
9. **Cooling speed:** To make the filament extruded and deposited during printing solidify faster, the layer fan can be used. This will reduce the likelihood of deformation. The layer fan, located in the printhead, will cool the filament as it exits the printer nozzle. The layer fan speed can be adjusted by assigning values between 0 and 255 for PWM (Pulse Width Modulation) control, which determine a fan motor speed from 0% to 100%, respectively.

To ensure that the printer is correctly levelled and calibrated, several printing tests were carried out. To this end, a rectilinear scaffold was printed, and the dimensions of each printed scaffold were checked to ensure that they corresponded to the dimensions established in the 3D model.

#### 2.2.2. Printing of Scaffolds

Once the 3D printer set-up was completed, the printing of scaffolds proceeded. A filament of polycaprolactone (PCL) (Facilan™ PCL100, 100% pure polycaprolactone homopolymer, molecular weight of 50.000 g/mol, 3D4makers.com, Haarlem, The Netherlands) was used for this purpose as it is a permeable biomaterial, which allows for a controlled drug release. It also provides shape memory, a low temperature melting point at around 60 °C, high elasticity at room temperature [28], and it is easy to print and blend with other polymers and loads [29].

It is advisable that the temperature and relative humidity of the room where printing takes place should not exceed the limits of 18–30 °C and 30–70% humidity. It is therefore of the utmost importance to not only control the environmental conditions during storage, but also the environmental conditions during printing [22]. Therefore, an environmental control was performed in each experiment to observe possible alterations during the printing of the scaffolds and the environmental conditions of temperature and humidity were noted for each print using a digital thermohygrometer.

**Figure 5:**
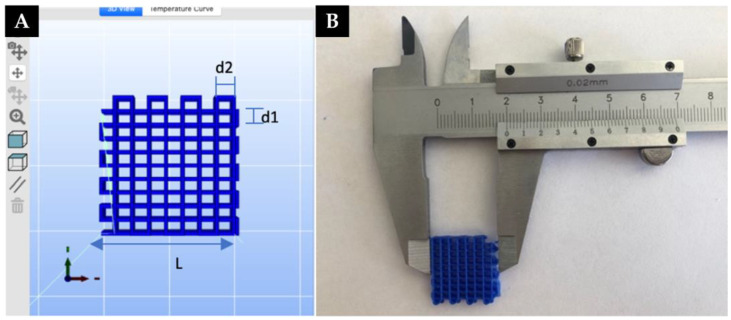
(**A**) Rectilinear geometric model of a scaffold generated in Repetier Host. (**B**) Printed scaffold measured with a caliper to test the shape and size fidelity. d1 is the distance between printed strands in the X-axis, and d2 in the Y-axis.

The printing of scaffolds was carried out considering the rectilinear geometrical model as shown in Figure 5. In order to facilitate the removal of the scaffold once the printing was finished, a magnetic flexible bed was used and 4-layer scaffolds were printed.

In Figure 5:

- L is the length of the scaffold
- d1 is the distance between printed strands in the X-axis
- d2 is the distance between printed filaments in the Y-axis.

It is necessary to optimize these parameters to obtain a scaffold with macropores as small as possible, without pore collapse. The selected values chosen for the experiments are summarized in Table 2.

Subsequently, several experiments were carried out by performing a sensitivity analysis to study the formation of microscopic fibers, modifying some printing parameters in a controlled way. These gave 5 different experimental studies:

1. Sensitivity analysis changing the extrusion volume through different values of the extrusion multiplier.
2. Sensitivity analysis of the printing speed.
3. Sensitivity analysis of the cool-down speed through the toggling of the extruder fan.
4. Performing minimum extrusion volume passes of filament on the printed scaffold without raising the layer in the Z-axis.
5. Reproducibility study once the printing conditions for microfiber formation have been identified.

The main objective of these experiments was focused on observing their influence on scaffold printing and the possible formation of microfibers. All the experiments were carried out considering a layer height of 0.20 mm for each of the 4 printed layers.

### 2.3. Microscopic Analysis of the Printed Scaffolds

Finally, to qualitatively evaluate the possible formation of microscopic fibers in the scaffolds printed by standard 3D printing, an analysis of images taken at the microscopic level was performed. For this purpose, a digital microscope (JIUSION 40 A 1000×, 8 LED USB 2.0) and Image J 1.8.0 (Image processing program [30]) were used.

A micrometric calibration ruler was used to calibrate the images taken with the digital microscope and evaluate the size of the printed filaments. A photo of the calibration ruler was taken with the digital microscope at the same distance as the photo taken of each scaffold. Subsequently, taking an average of twenty reference measurements (*n* = 20), the photo was calibrated with ImageJ.

## 3. Results

### 3.1. Extrusion Multiplier Sensitivity Analysis

To observe the influence of the extrusion volume on the possible formation of microfibers, the scaffold was printed at the manufacturer’s recommended printing speed (600 mm/min (10.0 mm/s)) considering different values of the extrusion multiplier (see Table 3).

**Figure 6:**
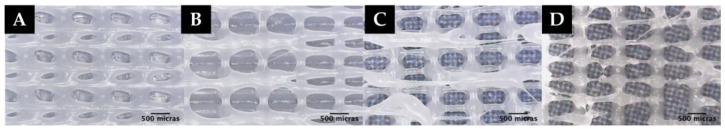
Microscopy images of experiment: (**A**) F1B, (**B**) F2B, (**C**) F3B, (**D**) F4B.

The environmental conditions at the time of printing (22.2 °C temperature and 59% humidity) are within the optimal range for printing. The results are shown in Figure 6.

As shown in Figure 6, when printing at a speed of 600 mm/min (10.0 mm/s) and an extrusion multiplier value of 1.0, no microfiber formation was observed. As the extrusion volume is decreased (0.60), random microfiber formation begins to be observed. It was also noticed that when making prints with the fan switched off, it takes up to 5 min for PCL to dry at room temperature. This is due to its low melting point.

### 3.2. Printing Speed Sensitivity Analysis

**Figure 7:**
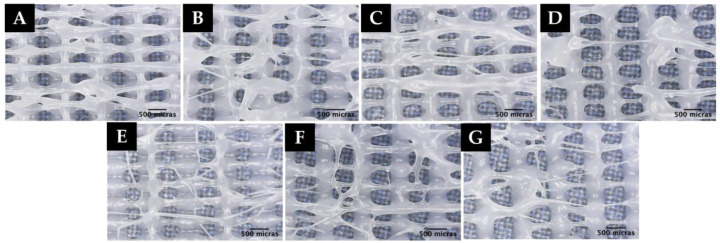
Microscopic images of experiment: (**A**) V1B, (**B**) V2B, (**C**) V3B, (**D**) V4B, (**E**) V5B, (**F**) V6B, (**G**) V7B.

Based on the results obtained in the sensitivity analysis of the extrusion volume, different experiments were carried out by printing the created scaffold model for an extrusion multiplier value of 0.60, 0.45, and 0.30 at different speeds, as shown in Table 4. The influence of the printing speed on the possible formation of microfibers was observed. For all the experiments, first layer printing speed was set at 600 mm/min (10.0 mm/s). The environmental conditions at the time of printing varied between 21 and 22 °C temperature and 59 and 61% humidity. The results of the printing are shown in Figure 7.

It should be noted that in the experiment performed with an extrusion multiplier of 0.30 (V8B), the first layer does not adhere to the hot bed due to the low amount of polymer coming out; therefore, no microscopic image of this experiment is included. As seen in Figure 7, printing at high speeds (1200 mm/min (20.0 mm/s)) and an extrusion multiplier below 0.6 results in random microfiber formation (V5B-V7B). However, it is also observed that when printing at high speeds, the printing quality decreases; therefore, lumps are formed in the printed scaffold, giving rise to an inhomogeneous morphology with respect to the dimensions of the macropores formed.

### 3.3. Sensitivity Analysis of the Cooling Rate

**Figure 8:**
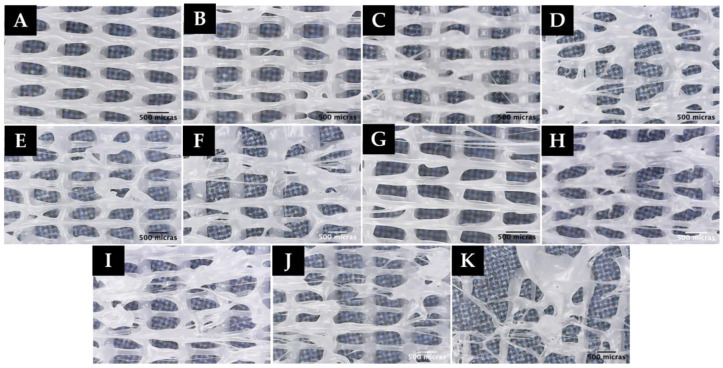
Microscopic images of experiments: (**A**) T1B, (**B**) T2B, (**C**) T3B, (**D**) T4B, (**E**) T5B, (**F**) T6B, (**G**) T7B, (**H**) T8B, (**I**) T9B, (**J**) T10B, (**K**) T11B.

Based on the results obtained in the previous analyses, different experiments were carried out by printing with the extruder fan on at different PWM values, as shown in Table 5. Also, the influence of the cooling rate on the scaffold printing and the formation of microfibers was observed. For all the experiments, first layer printing speed was set at 600 mm/min (10.0 mm/s). The environmental conditions at the time of printing varied between 21 and 22 °C temperature and 59 and 61% humidity. The printing results are shown in Figure 8.

As in the previous setup, and due to the small amount of material coming out from the nozzle, in experiments TB12 and TB13, the first layer does not adhere to the hot bed; thus, a printed scaffold could not be obtained under these conditions and, therefore, no microscopic image is included for these experiments.

As seen in the microscopic images (Figure 8), the use of the extruder fan helps the PCL to solidify faster, resulting in the formation of longer microfibers in the scaffold (T6B-T10B).

### 3.4. Further Study: Filament Passes without Raising the Layer in the Z-Axis

**Figure 9:**
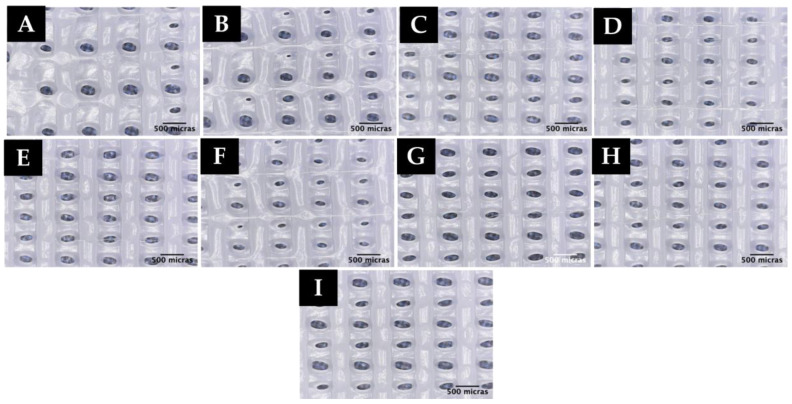
Microscopic image of experiments: (**A**) P1B, (**B**) P2B, (**C**) P3B, (**D**) P4B, (**E**) P5B, (**F**) P6B, (**G**) P7B, (**H**) P8B, (**I**) P9B.

To observe the effects of making minimum extrusion volume passes of filament on the printed scaffold without raising the layer in the Z-axis, 3 layers were printed with an extrusion multiplier equal to 1, the fan switched on at 100% (255 PWM) at a speed of 600 mm/min (10.0 mm/s), and with a layer height of 0.2 mm. For all the experiments, first layer printing speed was set at 600 mm/min (10.0 mm/s). Each filament pass was made on the last layer without raising in the Z-axis, while varying the extrusion volume and the printing speed (see Table 6). The environmental conditions during the prints performed in this sensitivity analysis were a temperature of 21 °C and 65% humidity. The printing results are shown in Figure 9.

Figure 9 shows that when the extrusion volume decreases (extrusion multiplier less than 0.20), microfibers begin to form in a high-speed manner (≥4200 mm/min (70.0 mm/s)), notably increasing when making passes with a minimum extrusion volume (extrusion multiplier 0.20) at a high speed (5000 mm/min (83.3 mm/s)).

**Figure 10:**
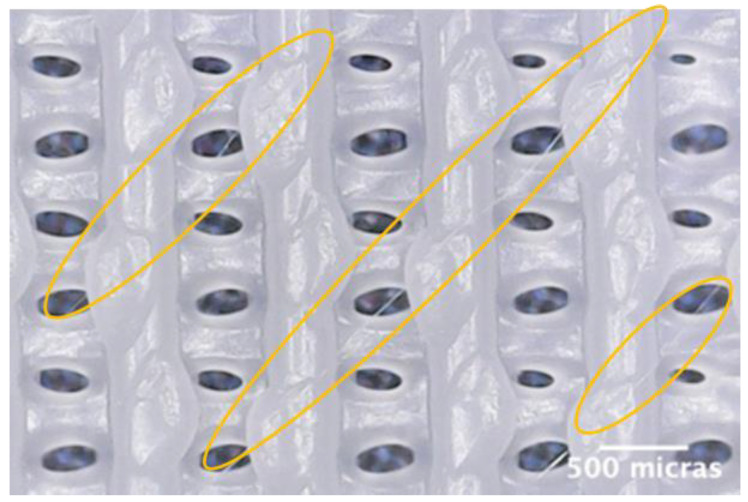
Microscopic images of experiment D1B.

Finally, an additional experiment (experiment D1B) was performed by printing a 3-layer scaffold at a printing speed of 600 mm/min (10.0 mm/s) and an extrusion multiplier of 1.00 and making diagonal passes on the third layer without raising in the Z-axis at a printing speed of 5000 mm/min (83.3 mm/s) and an extrusion multiplier of 0.20. Figure 10 shows the result of the printing at the microscopic level.

As shown in Figure 10, it is possible to create controlled microscopic fibers by combining the deposition routines generated in Python (G-code) and the tuning of printing parameters using standard 3D printing.

### 3.5. Reproducibility Study

**Figure 11:**
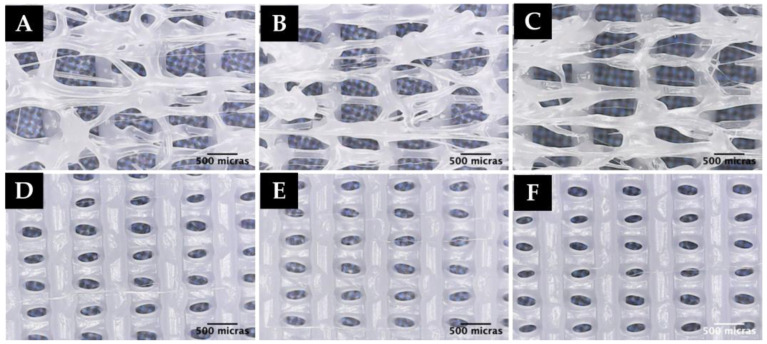
Microscope images of: (**A**–**C**) Reproducibility experiment T9B (4200 mm/min, PWM 255 and f = 0.45), (**D**–**F**) Reproducibility experiment P3B (4200 mm/min, PWM 255 and f = 0.20).

Finally, after finishing the sensitivity analyses and having confirmed that it is possible to obtain microfibers by standard 3D printers, a reproducibility study was carried out. For this purpose, 2 experiments performed during the sensitivity analyses (T9B and P3B) were selected and repeated in order to validate the reproducibility of the results. This study was performed under environmental conditions of 22 °C and 61% humidity. The results of this study are shown in Figure 11.

As can be seen in the above images, the random formation of microfibers under certain printing conditions observed in the sensitivity analyses and previously performed with PCL is reproducible.

## 4. Conclusions

The main objective of this work was the creation of reproducible microscopic internal fibers inside scaffolds printed by standard 3D printing. For this purpose, different experiments were carried out by depositing PCL and modifying the printing conditions (extrusion volume, printing, and cooling speeds), and subsequent microscopic analyses of their structures were performed. Through the sensitivity analysis performed on the manufacturing parameters and looking at the summary of the results obtained from the experiments performed (Appendix C), the following observations are worth mentioning as conclusions:

- The formation of controlled-shaped microfilaments (48 ± 12 µm, mean ± S.D.) is possible in scaffolds whose geometry has been generated through scripts based on primitive geometrical figures.
- With the layer fan off and at low speeds for material deposition (600 mm/min (10.0 mm/s)), random microfiber formation begins to be observed as the extrusion volume decreases below 0.60.
- When printing at high speeds with the extruder fan switched off, despite the formation of microfibers, the formation of lumps is observed. This influences the quality of the print, resulting in an inhomogeneous morphology of the formed macropores.
- The use of the extruder fan provides a faster drying of the PCL, reducing the formation of lumps or agglomerations of material during manufacturing and resulting in the formation of a greater quantity of microfibers.
- In general, it can be concluded that the optimal conditions for obtaining microfibers are at high speeds (4200–5000 mm/min (70–83.3 mm/s) and extrusion multiplier values between 0.60 and 0.45.
- It is possible to obtain controlled microfibers with the newly developed algorithms by making extruded filament passes without raising the layer in the Z-axis with an extrusion multiplier value of 0.2 and a print speed of 5000 mm/min (83.3 mm/s).

In short, this work has confirmed that it is possible to generate microfilaments by standard FDM 3D printing using the adequate slicing parameters and algorithms. Thus, it demonstrates the possibility of creating scaffolds with macroscopic pores and microscopic niches for possible medical applications using low-cost, accessible methods.

## Figures and Tables

**Table 1:** Configuration of the FDM 3D printer “Artillery Genius” selected for the study [23].

CHARACTERISTICS	VALUES
Maximum printing speed	150 mm/s
Maximum travel speed	250 mm/s
X, Y, Z resolution	0.05 mm, 0.05 mm, 0.10 mm
Filament diameter	1.75 mm
Nozzle diameter	0.40 mm

**Table 2:** Reference values used as a model base for the study to generate rectilinear patterns with PCL.

PRINTING CONDITIONS	VALUES
d1	1.0 mm
d2	2.5 mm
L	20.0 mm
Layer height	0.20 mm
Hot bed temperature	40 °C
Extruder temperature	150 °C
Extrusion multiplier	1.00
Printing speed	600 mm/min (10.0 mm/s)
Fan (Switch on/off)	Off

**Table 3:** Printing parameters of experiments F1B, F2B, F3B, and F4B.

EXPERIMENT	F1B	F2B	F3B	F4B
PRINTING CONDITIONS	VALUES			
Extrusion multiplier	1.00	0.60	0.45	0.30

**Table 4:** Printing parameters of experiments V1B-V8B.

EXPERIMENT	V1B	V2B	V3B	V4B
PRINTING CONDITIONS	VALUES			
Extrusion multiplier	0.60	0.60	0.45	0.45
Printing speed	5000 mm/min (83.3 mm/s)	6000 mm/min (100.0 mm/s)	1200 mm/min (20.0 mm/s)	3000 mm/min (50.0 mm/s)
**EXPERIMENT**	**V5B**	**V6B**	**V7B**	**V8B**
**PRINTING** **CONDITIONS**	**VALUES**			
Extrusion multiplier	0.45	0.60	0.45	0.30
Printing speed	4200 mm/min (70.0 mm/s)	5000 mm/min (83.3 mm/s)	6000 mm/min (100.0 mm/s)	1200 mm/min (20.0 mm/s)

**Table 5:** Printing parameters of experiments T1B-T13B.

EXPERIMENT	T1B	T2B	T3B	T4B
PRINTING CONDITIONS	VALUES			
Extrusion multiplier	0.60	0.60	0.60	0.45
Printing speed	600 mm/min (10.0 mm/s)	5000 mm/min (83.3 mm/s)	5000 mm/min (83.3 mm/s)	6000 mm/min (100.0 mm/s)
PWM	255 (100 %)	150 (58.8 %)	255 (100 %)	50 (19.6%)
**EXPERIMENT**	**T5B**	**T6B**	**T7B**	**T8B**
**PRINTING CONDITIONS**	**VALUES**			
Extrusion multiplier	0.60	0.60	0.45	0.45
Printing speed	6000 mm/min (100.0 mm/s)	6000 mm/min (100.0 mm/s)	600 mm/min (10.0 mm/s)	3000 mm/min (50.0 mm/s)
PWM	150 (58.8 %)	255 (100 %)	255 (100 %)	255 (100 %)
**EXPERIMENT**	**T9B**	**T10B**	**T11B**	**T12B**
**PRINTING CONDITIONS**	**VALUES**			
Extrusion multiplier	0.45	0.45	0.45	0.45
Printing speed	4200 mm/min (70.0 mm/s)	5000 mm/min (83.3 mm/s)	6000 mm/min (100.0 mm/s)	6000 mm/min (100.0 mm/s)
PWM	255 (100 %)	255 (19.6%)	50 (100 %)	255 (19.6%)
**EXPERIMENT**	**T13B**			
**PRINTING CONDITIONS**	**VALUES**			
Extrusion multiplier	0.30			
Printing speed	600 mm/min (10.0 mm/s)			
PWM	255 (100 %)			

**Table 6:** Printing parameters of experiments P1B-P9B.

EXPERIMENT	P1B	P2B	P3B	P4B
PRINTING CONDITIONS	VALUES			
Extrusion multiplier	0.40	0.20	0.20	0.20
Printing speed	6000 mm/min (100.0 mm/s)	600 mm/min (10.0 mm/s)	4200 mm/min (70.0 mm/s)	5000 mm/min (83.3 mm/s)
**EXPERIMENT**	**P5B**	**P6B**	**P7B**	**P8B**
**PRINTING CONDITIONS**	**VALUES**			
Extrusion multiplier	0.20	0.10	0.10	0.10
Printing speed	6000 mm/min (100.0 mm/s)	600 mm/min (10.0 mm/s)	4200 mm/min (70.0 mm/s)	5000 mm/min (83.3 mm/s)
**EXPERIMENT**	**P9B**			
**PRINTING CONDITIONS**	**VALUES**			
Extrusion multiplier	0.10			
Printing speed	6000 mm/min (100.0 mm/s)			

## Data Availability

Not applicable.

## Appendix A

**Python script for the rectilinear geometric model.**

As an example, the Python script written for the rectilinear geometric model is shown below.

*„„„* *x0,y0* *_________________* *________________| ^* *|________________ |* *________________| | L* *|________________ |* *________________|v* *<----------------------->* *L* *„„„* *# Import numpy library (numerical computation) and matpotlib (generate plots from data in lists and vectors)* *import numpy as np* *import matplotlib.pyplot as plt* *# Definition of variables* *# independent variables* *L = 30 # scaffold length (mm)* *D = 1.75 # filament diameter (mm)* *d = 0.400 # extruder nozzle diameter (mm)* *dist1= 1 # distance between lines of the first layer (mm)* *dist2 = 2 # line spacing of second layer (mm)* *nz = 4 # number of layers* *ts = 1200 # traverse speed (mm/min)* *fr = 800 # printing speed (mm/min)* *flow = 1 # extrusion multiplier* *E_extruded = 0 # initialization of extruded length (mm)* *h = 0.2 # layer height (mm)* *# Dependent variables* *n1 = int(L/dist1) # number of turns per horizontal layer* *n2 = int(L/dist2) # number of turns per vertical layer* *w = 2*h # extrusion width* *A= (w-h)*h + pi*((h/2)**2) # cross section of extruded filament* *# EXTRUSION FUNCTION* *# extrusion volume calculation for a given length (L)* *def extrusion(L):* *ext = L*(A*4)/(pi*(D**2)) #source: Slic3r* *return ext* *# FIRST LAYER* *# assign vector (x, y, z), initial position* *P0 = [100-L/2, 100-L/2, h]* *xpos1 = [] #1D array—storage of x values for g-code* *ypos1 = [] #1D array—storage of x values for the g-code* *extr_l = [] #1D array—storage of the extrusion values for the g-code* *# GENERATION OF XY POSITIONS* *ypos1.append(P0[1]**) # initial position Y* *for j in range (0,int(n1/2)): # generate positions in X for n1 laps* *xpos1.append(P0[0]**)* *xpos1.append(P0[0] + L)* *xpos1.append(P0[0] + L)* *xpos1.append(P0[0])* *for k in range (1,2*n1-1): #generate Y positions for n1 turns* *if (k % 2) == 0:* *ypos1.append(ypos1[-1]) # Y position (k= even number).* *else:* *ypos1.append(ypos1[-2] + dist1) # Y position (k= odd number).* *# G-Code generation and text file creation* *# Write a .txt file with the values obtained and the G-code format for each position:* *file = open(“gcode.txt”, “w+”)* *extr_l.append(0)* *print(‘G0 F{:.0f} X{:.3f} Y{:.3f} Z{:.3f}’.format(ts, P0[0]**, P0[1]**, P0[2]))* *for o in range(1,len(xpos1)):* *extr_l.append(np.abs((xpos1[o]-xpos1[o-1]) + (ypos1[o]-ypos1[o-1])))* *E_extruded = E_extruded + extrusion(extr_l[o])*flow* *print(‘G1 F{:.0f} X{:.3f} Y{:.3f} E{:.3f}’.format(fr, xpos1[o], ypos1[o], E_extruded))* *file.write(‘G1 F{:.0f} X{:.3f} Y{:.3f} E{:.3f} E{:.3f}’.format(fr, xpos1[o], ypos1[o], E_extruded))* *# Save and close file* *file.close* *# Generate XY positions for a scaffold with ‘nz’ layers* *for i in range (1,nz):* *# Generate XY positions for even layers.* *if (i % 2) = = 0:* *P2 = [100-L/2, 100-L/2, (i + 1)*h]* *xpos2 = []* *ypos2 = []* *ypos2.append(P2[1]**)* *ypos2.append(P2[1]**)* *for j in range (0,int(n1/2)):* *xpos2.append(P2[0]**)* *xpos2.append(P2[0] + L)* *xpos2.append(P2[0] + L)* *xpos2.append(P2[0]**)* *for k in range (1,2*n1-1):* *if (k % 2) == 0:* *ypos2.append(ypos2[-1])* *else:* *ypos2.append(ypos2[-2] + dist1)* *file = open(„gcode.txt”,”w+”) # ‘w’ for write, the ‘ + ’ to create the file if it does not exists* *extr_l.append(0)* *print(‘G0 F{:.0f} X{:.3f} Y{:.3f} Z{:.3f}’.format(ts, P2[0]**, P2[1]**, P2[2]))* *for o in range(1,len(xpos2)):* *extr_l.append(np.abs((xpos2[o]-xpos2[o-1]) + (ypos2[o]-ypos2[o-1])))* *E_extruded = E_extruded + extrusion(extr_l[o])*flow2* *print(‘G1 F{:.0f} X{:.3f} Y{:.3f} E{:.3f}’.format(fr, xpos2[o], ypos2[o], E_extruded))* *file.write(‘G1 F{:.0f} X{:.3f} Y{:.3f} E{:.3f}\n’.format(fr, xpos2[o], ypos2[o], E_extruded))* *file.close* *# Generate XY positions for odd layers* *else:* *P3 = [100-L/2, 100-L/2, (i + 1)*h] #100-L/2.5* *xpos3 = []* *ypos3 = []* *xpos3.append(P3[0])* *for j in range (0,2*n2-1)* *if (j % 2) == 0:* *xpos3.append(xpos3[-1])* *else:* *xpos3.append(xpos3[-2] + dist2)* *for k in range (0,int(n2/2)):* *ypos3.append(P3[1]**)* *ypos3.append(P3[1] + L)* *ypos3.append(P3[1] + L)* *ypos3.append(P3[1]**)* *file = open(„gcode.txt”,”w+”) # ‘w’ for write, the ‘+’ to create the file if it does not exists* *extr_l.append(0)* *print(‘G0 F{:.0f} X{:.3f} Y{:.3f} Z{:.3f}’.format(ts, P3[0]**, P3[1]**, P3[2]**)* *for o in range(1,len(xpos3)):* *extr_l.append(np.abs((xpos3[o]-xpos3[o-1]) + (ypos3[o]-ypos3[o-1])))* *E_extruded = E_extruded + extrusion(extr_l[o])*flow2* *print(‘G1 F{:.0f} X{:.3f} Y{:.3f} E{:.3f}’.format(fr, xpos3[o], ypos3[o], E_extruded))* *file.write(‘G1 F{:.0f} X{:.3f} Y{:.3f} E{:.3f}\n’.format(fr, xpos3[o], ypos3[o], E_extruded))* *file.close* *# GENERATE PLOT* *ax = plt.figure().gca()* *plt.axis(‘square’)* *plt.ylim(80, 120)* *plt.xlim(80, 120)* *#plot the final g-code values (green, red, blue))* *ax.plot(xpos3,ypos3,’g’)* *ax.plot(xpos1,ypos1,’b’)* *plt.show()*

## Appendix B

G-CODE SCRIPT

As an example, the following is the G-code script generated for 3D printing a 1-layer scaffold created from the rectilinear geometric model (L = 30 mm, d1 = 1 mm).

*M140 S40; —ACTIVATE HOT BED AND EXTRUDER TEMPERATURE—SET THE TEMPERATURE OF THE HOT BED AND THE EXTRUDER—SET THE TEMPERATURE OF THE EXTRUDER—SET THE TEMPERATURE OF THE EXTRUDER* *M140 S40 ; sets the hot bed temperature to 40 °C (do not wait to reach the value)* *M104 S150 ; sets extruder temperature to 150 °C (do not wait for the value to be reached)* *M105 ; requests the extruder temperature value* *M190 S40 ; sets the hot bed temperature to 40 °C (wait until the value is reached)* *M105 ; requests extruder temperature value* *M109 S150 ; sets extruder temperature to 150 °C (wait until value is reached)* *M82 ; makes the extruder interpret the extrusion as absolute positions* *-- START G-CODE --* *M302 S120 ; allows for printing at temperatures lower than 170 °C* *G21 ; sets the units in millimeters* *G90 ; sets the extruder position as absolute position* *M106 S0 ; sets the fan speed (PWM 0—off)* *G28 X0 Y0 ; moves the extruder to the origin (X/Y Home)* *M117 ; purges the extruder* *G92 E0 ; restarts the extruder* *G28 Z0 ; moves the extruder to the origin (Z Home)* *G1 Z15.0 F2400 ; moves the extruder to the Z 15.0 mm position* *G92 E0 ; does not extrude the filament on the move* *G1 E1 F200 ; extrudes 1mm of filament* *G1 E1 F200 ; —end of START G-CODE—* *; PRINTING LAYER_1 (Code generated in Python)* *; fast motion (traverse speed F mm/min; Position XYZ (mm))* *G0 F800 X85.000 Y85.000 Z0.200* *; controlled motion (printing speed F (mm/min); XY position (mm); extruded filament E (mm))* *G1 F600 X115.000 Y85.000 E0.891* *G1 F600 X115.000 Y86.000 E0.920* *G1 F600 X85.000 Y86.000 E1.811* *G1 F600 X85.000 Y87.000 E1.841* *G1 F600 X115.000 Y87.000 E2.732* *G1 F600 X115.000 Y88.000 E2.761* *G1 F600 X85.000 Y88.000 E3.652* *G1 F600 X85.000 Y89.000 E3.682* *G1 F600 X115.000 Y89.000 E4.572* *G1 F600 X115.000 Y90.000 E4.602* *G1 F600 X85.000 Y90.000 E5.493* *G1 F600 X85.000 Y91.000 E5.523* *G1 F600 X115.000 Y91.000 E6.413* *G1 F600 X115.000 Y92.000 E6.443* *G1 F600 X85.000 Y92.000 E7.334* *G1 F600 X85.000 Y93.000 E7.363* *G1 F600 X115.000 Y93.000 E8.254* *G1 F600 X115.000 Y94.000 E8.284* *G1 F600 X85.000 Y94.000 E9.175* *G1 F600 X85.000 Y95.000 E9.204* *G1 F600 X115.000 Y95.000 E10.095* *G1 F600 X115.000 Y96.000 E10.125* *G1 F600 X85.000 Y96.000 E11.015* *G1 F600 X85.000 Y97.000 E11.045* *G1 F600 X115.000 Y97.000 E11.936* *G1 F600 X115.000 Y98.000 E11.966* *G1 F600 X85.000 Y98.000 E12.856* *G1 F600 X85.000 Y99.000 E12.886* *G1 F600 X115.000 Y99.000 E13.777* *G1 F600 X115.000 Y100.000 E13.806* *G1 F600 X85.000 Y100.000 E14.697* *G1 F600 X85.000 Y101.000 E14.727* *G1 F600 X115.000 Y101.000 E15.618* *G1 F600 X115.000 Y102.000 E15.647* *G1 F600 X85.000 Y102.000 E16.538* *G1 F600 X85.000 Y103.000 E16.568* *G1 F600 X115.000 Y103.000 E17.458* *G1 F600 X115.000 Y104.000 E17.488* *G1 F600 X85.000 Y104.000 E18.379* *G1 F600 X85.000 Y105.000 E18.409* *G1 F600 X115.000 Y105.000 E19.299* *G1 F600 X115.000 Y106.000 E19.329* *G1 F600 X85.000 Y106.000 E20.220* *G1 F600 X85.000 Y107.000 E20.249* *G1 F600 X115.000 Y107.000 E21.140* *G1 F600 X115.000 Y108.000 E21.170* *G1 F600 X85.000 Y108.000 E22.061* *G1 F600 X85.000 Y109.000 E22.090* *G1 F600 X115.000 Y109.000 E22.981* *G1 F600 X115.000 Y110.000 E23.011* *G1 F600 X85.000 Y110.000 E23.901* *G1 F600 X85.000 Y111.000 E23.931* *G1 F600 X115.000 Y111.000 E24.822* *G1 F600 X115.000 Y112.000 E24.852* *G1 F600 X85.000 Y112.000 E25.742* *G1 F600 X85.000 Y113.000 E25.772* *G1 F600 X115.000 Y113.000 E26.663* *G1 F600 X115.000 Y114.000 E26.692* *G1 F600 X85.000 Y114.000 E27.583* *; —END G-CODE—* *M104 S0; sets the extruder temperature to zero (off)* *G91; sets the extruder position as relative position* *G1 E-2 F600 ; retracts the filament to release pressure* *G1 Z10 ; moves the extruder to position Z 10 mm* *G90; sets the extruder position as absolute position* *M84; switches off the motor* *; —END G-CODE—*

## Appendix C

**Summary of results**

**Table A1:** Summary of results with the fan switched off.

FAN OFF								
**Extrusion Factor**	**Printing Speed (mm/min)**							
	300	600	800	1200	3000 *	4200 *	5000 *	6000 *
1								
0.6								
0.45								
0.3								

* First layer print at 600 mm/min. Color legend: (Red) No microfiber formation is observed; (Light green) Random microfiber formation; (Grass green) Random microfiber formation increases significantly; (Blue) Microfiber formation with greater length and homogenous distribution; (Yellow) Poor adhesion/poor print quality.

**Table A2:** Summary of results with the fan switched on.

FAN ON								
**Extrusion Factor**	**Printing Speed (mm/min)**							
	300	600	800	1200	3000 *	4200 *	5000 *	6000 *
1								
0.6								
0.45								
0.3								

* First layer print at 600 mm/min. Color legend: (Red) No microfiber formation is observed; (Light green) Random microfiber formation; (Grass green) Random microfiber formation increases significantly; (Blue) Microfiber formation with greater length and homogenous distribution; (Yellow) Poor adhesion/poor print quality.

**Table A3:** Summary of results with the fan switched on, doing passes in the Z-axes with a low extrusion factor.

PASSES IN THE Z-AXES (FAN ON)								
**Extrusion Factor**	**Printing Speed (mm/min)**							
	300 **	600 **	800 **	1200 **	3000 **	4200 **	5000 **	6000 **
0.4								
0.2								
0.1								

** Printing speed of the first three layers: 600 mm/min. Color legend: (Red) No microfiber formation is observed; (Light green) Random microfiber formation; (Grass green) Random microfiber formation increases significantly; (Blue) Microfiber formation with greater length and homogenous distribution; (Yellow) Poor adhesion/poor print quality.
